# Supplementary material for: Macrophage morphological plasticity and migration is Rac signalling and MMP9 dependant
Source: Sci Rep. 2021 May 12;11:10123. doi: 10.1038/s41598-021-88961-7 (PMC8115330; doi:10.1038/s41598-021-88961-7)
Supplement: Supplementary file 1 — Supplementary Information 1. [file 41598_2021_88961_MOESM1_ESM.pdf]

Supplementary Information

to

Macrophage morphological plasticity and migration is Rac signalling and MMP9 dependant

Jana Travnickova[1,2], Sandra Nhim[1], Naoill Abdellaoui[1], Farida Djouad[3], Maï Nguyen-Chi[4,]
Andrea Parmeggiani[1,5] and Karima Kissa[1,*]

[1] LPHI, CNRS, INSERM, Univ Montpellier, Montpellier, France - Emergence of haematopoietic stem cells and cancer
[2] MRC Human Genetics Unit, and CRUK Edinburgh Centre, MRC Institute of Genetics and Molecular Medicine, Western General Campus, University of Edinburgh, Edinburgh EH4 2XU, UK
[3]IRMB, Inserm U1183, CHU Saint Eloi- Cellules souches, Blastème et Régénération, F-34295, Montpellier, France
[4] LPHI, CNRS, INSERM, Univ Montpellier, Montpellier, France - Mise en place de l'immunité et inflammation
[5] Laboratoire Charles Coulomb, CNRS, Univ Montpellier, Montpellier, France

***** Correspondence: karima.kissa-marin@umontpellier.fr
[2] Current adress

Supplementary Table S1. Measured average values (± s.e.m.) of shape descriptors for each shape subgroup. N = 20 embryos.

Shape	Circularity	Roundness	Elongation factor
**Round**	0.19 ± 0.01	0.65 ± 0.04	1.55 ± 0.10
**Amoeboid**	0.07 ± 0.004	0.49 ± 0.01	2.04 ± 0.05
**Star-like**	0.07 ± 0.004	0.70 ± 0.01	1.56 ± 0.07
**Elongated**	0.07 ± 0.005	0.35 ± 0.01	3.45 ± 0.14

Supplementary Figure S1

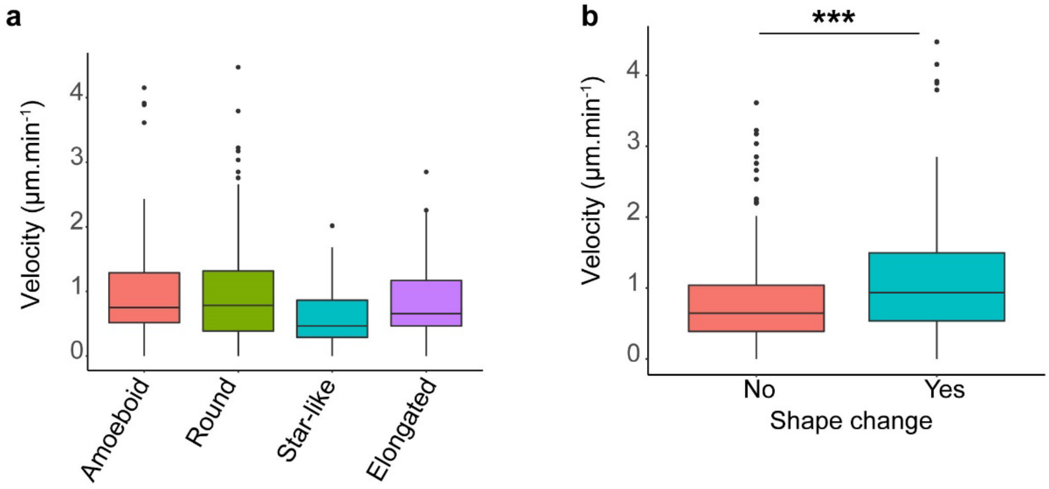

Supplementary Figure S1. Correlation between cell shape and instantaneous velocity. **(a)** Box plot shows the comparable macrophage instantaneous velocity for each shape. **(b)** Box plot shows significantly higher speed of macrophages actively changing the shape. *** $p<0.001$, Mann-Whitney test. For each macrophage, and at each time point of imaging acquisition (every minute), the distance travelled by each macrophage was measured.

Supplementary Figure S2

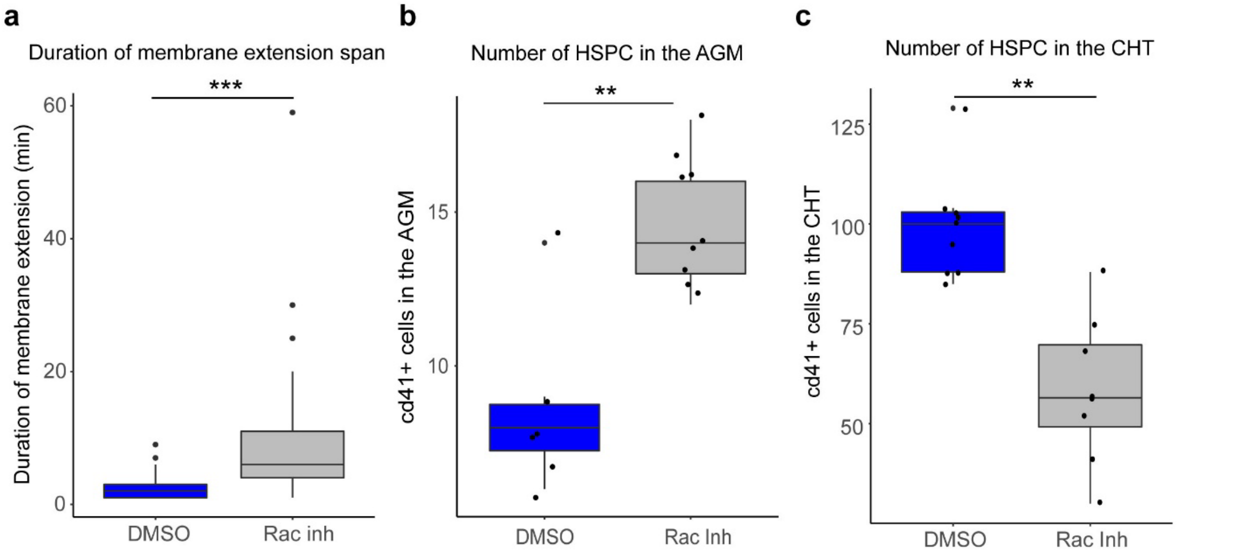

Supplementary Figure S2. Effect of Rac inhibition on macrophage membrane extensions and HSPC mobilisation and colonisation.

(a) Graph shows the macrophage membrane single extension span between control and Rac inhibition. *** $p<0.001$, n=45 extensions. **(b-c)** Box dot plots show number of HSPC in the AGM **(b)** or CHT **(c)** of embryos treated with Rac inhibitor compared to DMSO treated control. ** $p < 0.01$, each dot represents one embryo. All tests: Mann-Whitney test.

Video 1. Macrophage 3D migration in the AGM during haematopoiesis.

Representative time-lapse colour-depth projections of *Tg(mpeg1:mCherry*) embryo at 46 hpf illustrate macrophages migration occurring in a non-directional manner and through different depth by appropriately changing colour . Image stacks were acquired every minute over 60 minutes at a 1 µm interval with 1024x256 pixel resolution using the LSM510 Zeiss confocal microscope equipped with a 40x water immersion objective. Scale bar 30 µm, time code in hours and minutes.

Video 2. The migratory behaviour of macrophage changes after Rac inhibition.

Combined representative time-lapse colour-depth projections of *Tg(mpeg1:mCherry)* embryos at 46 hpf draws a comparison between macrophage migration in DMSO-treated (control, top) embryos and that of Rac-inhibitor (Rac inh, bottom) treated embryos. Rac-inhibited macrophages display slower migration modes. They change shapes and migration direction less often, and form very long membrane extensions. Image stacks were acquired every minute over 60 minutes at a 1 µm interval with 1024x256 pixel resolution using the LSM510 Zeiss confocal microscope equipped with a 40x water immersion objective. Scale bar 30 µm, time code in hours and minutes.

Video 3. MMP inhibition induces mesenchymal-amoeboid transition of macrophage migration.

Combined representative time-lapse colour-depth projections of *Tg(mpeg1:mCherry*) embryos at 46 hpf draw a comparison between macrophage migration in DMSO-treated (control, top) and MMP-2 and 9 inhibitor (MMP inh, bottom) treated embryos. MMP-inhibited macrophages migrate faster, adopt a round shape, change the depth of their displacement less often and migrate partially

inside the bloodstream. Image stacks were acquired every minute over 60 minutes at 1 µm interval with 1024x256 pixel resolution using the LSM510 Zeiss confocal microscope equipped with a 40x water immersion objective. Scale bar 30 µm, time code expressed in hours and minutes.
